# Epidemiologic characteristics of death by burn injury from 2000 to 2009 in Colombia, South America: a population-based study

**DOI:** 10.1186/s41038-016-0033-0

**Published:** 2016-03-16

**Authors:** Norberto Navarrete, Nelcy Rodriguez

**Affiliations:** 1 Burn Intensive Care Unit, Simón Bolívar Hospital, Bogotá, Colombia; 2 Clinical Epidemiology and Biostatistics Department, Javeriana University, Bogotá, Colombia; 3 Department of Statistics, National University of Colombia, Bogotá, Colombia

## Abstract

**Background:**

Burns are one of the most severe traumas that an individual can suffer. The World Health Organization (WHO) affirms that injuries related to burns are a global public health problem mainly in low- and middle-income countries. The first step towards reducing any preventable injury is based on accurate information. In Colombia, the basic epidemiological characteristics of burn injuries are unknown. The objectives were establishing the causes, high-risk populations, mortality rate, and tendencies of burn deaths.

**Methods:**

Observational, analytical, population-based study based on official death certificate occurred between 2000 and 2009. All codes of the International Classification of Diseases-10th Revision (ICD-10) related to burns were included. The mortality rates were standardized using the WHO world average age weights 2000–2025. To determine the tendency, an average annual percent change (AACP) was calculated.

**Results:**

A total of 5448 deaths due to burns were identified; 78.4 % were men. The crude and adjusted burn mortality rate was 1.270 and 1.302 per 100,000, respectively. The AACP was −5.25 %. Electrical injury caused the greatest number of deaths (49.5 %), followed by fire and lightning injuries. A total of 1197 (22.1 %) children were under 15 years old. The causes of deaths were different among age groups. 59.4 % deaths occurred outside health institutions.

**Conclusions:**

This study is a first step in identifying the main causes of death and groups with higher mortality rates. Electricity is the main cause of deaths due to burn injury. Further research is required in order to generate awareness among government and community for reducing the number of injuries and burn deaths in our country.

## Background

Traumatic injuries represent one of the most important public health problems that both developing and industrialized countries face. In Colombia in the year 2009, there were 35,430 deaths due to external causes or trauma, which account for 18.0 % of all deaths [1]. Burned patients present one of the most severe traumas to which an individual can be exposed and produce a significant morbidity and mortality in developed or developing countries at all ages [2].

Burns are not just a medical problem. Both the patient and the family face physical and psychological problems that go beyond the period of hospitalization [3–6]. The society also shares these effects in terms of disease burden, not only for the high costs of the treatment but also because fire-related burns are among the leading causes of disability-adjusted life years (DALYs) [7, 8].

Like the vast majority of traumatic injuries, burn injuries may be preventable [9–11]; epidemiological studies must be performed in order to establish special information to be provided as the basis for prevention programs, for their design and implementation. This paper presents the first general approach to the problem of burn injuries in Colombia, which may allow the effective design and implementation of prevention programs, and therefore have an impact in the reduction of the mortality rate and sequelae of patients with burns.

## Methods

During the middle of 2011, an academic project was begun to obtain epidemiological information regarding thermal injuries in our country (*PREVER*: Register and Evaluation of Electricity and Lightning Injuries Program), with the support of two scientific societies. We requested developing a customized process for deaths caused by burns, with codes according to the International Classification of Diseases-10th Revision (ICD-10) of the World Health Organization (WHO) (Table 1), from 1 January 2000 to 31 December 2009. Official data was collected by the National Administrative Department of Statistics *(Departamento Administrativo Nacional de Estadistica—DANE*) on vital statistics—death certificates. In Colombia, a death certificate must be completed after conducting the autopsy process in all accidental deaths.

**Table 1:** International classification of disease (ICD-10) codes to burn agent

Code	Definition	Category
W85–W87	Exposure to electric current	Electric
X00–X09	Exposure to smoke, fire, and flames	Fire
X10	Contact with hot drinks, food, fats, and cooking oils	Hot liquids
X11	Contact with hot tap water	Hot liquids
X12	Contact with other hot fluids	Hot liquids
X13	Contact with steam and hot vapors	Hot gases
X14	Contact with hot air and gases	Hot gases
X15	Contact with hot household appliances	Hot solid
X16	Contact with hot heating appliances, radiators, and pipes	Hot solid
X17	Contact with hot engines, machinery, and tools	Hot solid
X18	Contact with other hot metals	Hot solid
X19	Contact with other and unspecified heat and hot substances	Unspecified
X33	Victim of lightning	Electric
X76	Intentional self-harm by smoke, fire, and flames	Self-harm
X77	Intentional self-harm by steam, hot vapors, and hot objects	Self-harm

We conducted an observational, descriptive, retrospective analysis. The data described has to be considered as population-based rather than hospital-based study. Data collected includes sociodemographic standard information (age, gender, area and site of death, marital status, educational level, and social security regime). Mortality rates are expressed as deaths per 100,000 people using official estimated population by DANE [1]. The rates were age-standardized using the direct method with 5-year age categories and were calculated using the WHO world average age weights 2000–2025. We performed an average annual percent change (AAPC), to determine the tendency of the adjusted mortality rate.

All statistical calculations were done using the program Stata, 11th version, educational license (StataCorp, College Station, TX) except AAPC, which was done on Microsoft Excel (2010).

In Colombia, resolution 8430 of 1993 defines these types of studies as those with “no risk” since they do not identify the people who are object of the study or treat sensitive issues. Also, no physiological variables are manipulated or patients accessed directly. Thus, no previous evaluation from the ethics committee was requested for using this database.

## Results

**Fig. 1:**
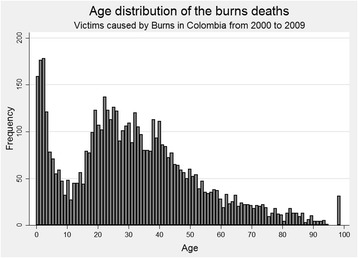
Age distribution of cases in Colombia fatalities by burns during the years 2000–2009. The *bar* at age 99 is for patients with indeterminate age during the processing of the death certificate

In total, 5448 deaths due to burns were identified from the death certificates; 4270 (78.4 %) were men; the male to female ratio was 3.6:1; age was unknown for 31 of the deaths. The age range was from 3 days to 95 years, median was 29 years (interquartile range (IQR) 17–43). Age distribution is shown in Fig. 1. By gender, males had a mean age of 31.8 years (IQR 19–43) and females a mean age of 28.6 (IQR 4–46). In women of childbearing age, there were 19 (3.4 %) cases of pregnancy who died from burns. At the time of signing the death certificate, it was unknown whether 249 (44.8 %) of the women from this group were pregnant or not. Although in Colombia, people under 18 are considered underage, hospitals usually regard children for the pediatric population until they are 14 years old. A total of 1197 children under 15 years old died (22.1 %) with median age of 3 years (IQR 1–8); 735 (61.4 %) were men. This gives a male to female ratio of 1.6:1 among the pediatric population.

**Fig. 2:**
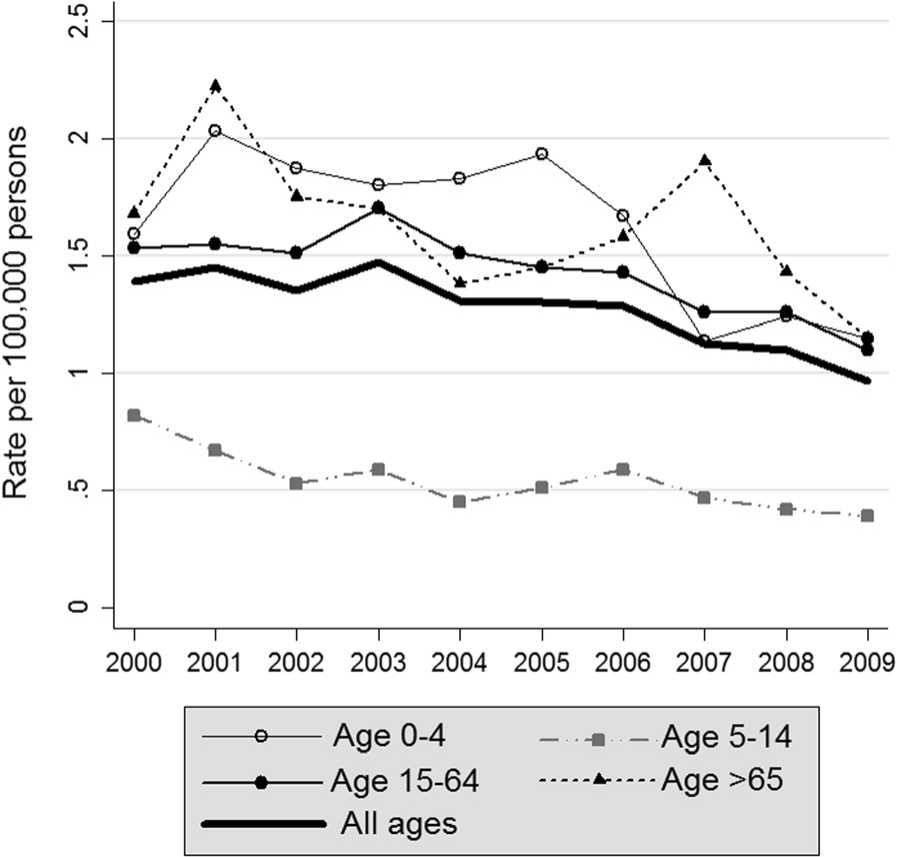
Annual distribution and non-standardized rate of fatal burns in Colombia in the years 2000–2009

The burn mortality rate in Colombia is 1.270 per 100,000 per year during the study period (Table 2). The adjusted mortality rate was 1.302 per 100,000 per year. There was a progressive decline in mortality from burns in the course of the years of the study. The average percent mortality change for burn deaths was −5.25 %. Populations with higher mortality rates are those under 5 years and over 65 years old (Fig. 2). The mortality rate for male was 2.029 and for females 0.546.

**Table 2:** Crude and standardized rate of fatal burns in Colombia in the years 2000–2009

Year	Deaths	Estimated population	Crude mortality rate/100,000	Adjusted mortality rate/100,000	Confidence interval
2000	560	40,295,563	1.390	1.400	[1.438–1.463]
2001	591	40,813,541	1.448	1.455	[1.487–1.512]
2002	559	41,328,824	1.353	1.362	[1.386–1.410]
2003	617	41,848,959	1.474	1.474	[1.503–1.528]
2004	554	42,368,489	1.308	1.317	[1.329–1.352]
2005	558	42,888,592	1.301	1.308	[1.305–1.327]
2006	559	43,405,956	1.288	1.292	[1.306–1.328]
2007	495	43,926,929	1.127	1.138	[1.153–1.174]
2008	488	44,451,147	1.098	1.102	[1.114–1.134]
2009	436	44,978,832	0.969	0.978	[0.969–0.987]
Total	5417	42,630,683	1.270	1.302	[1.299–1.306]

**Fig. 3:**
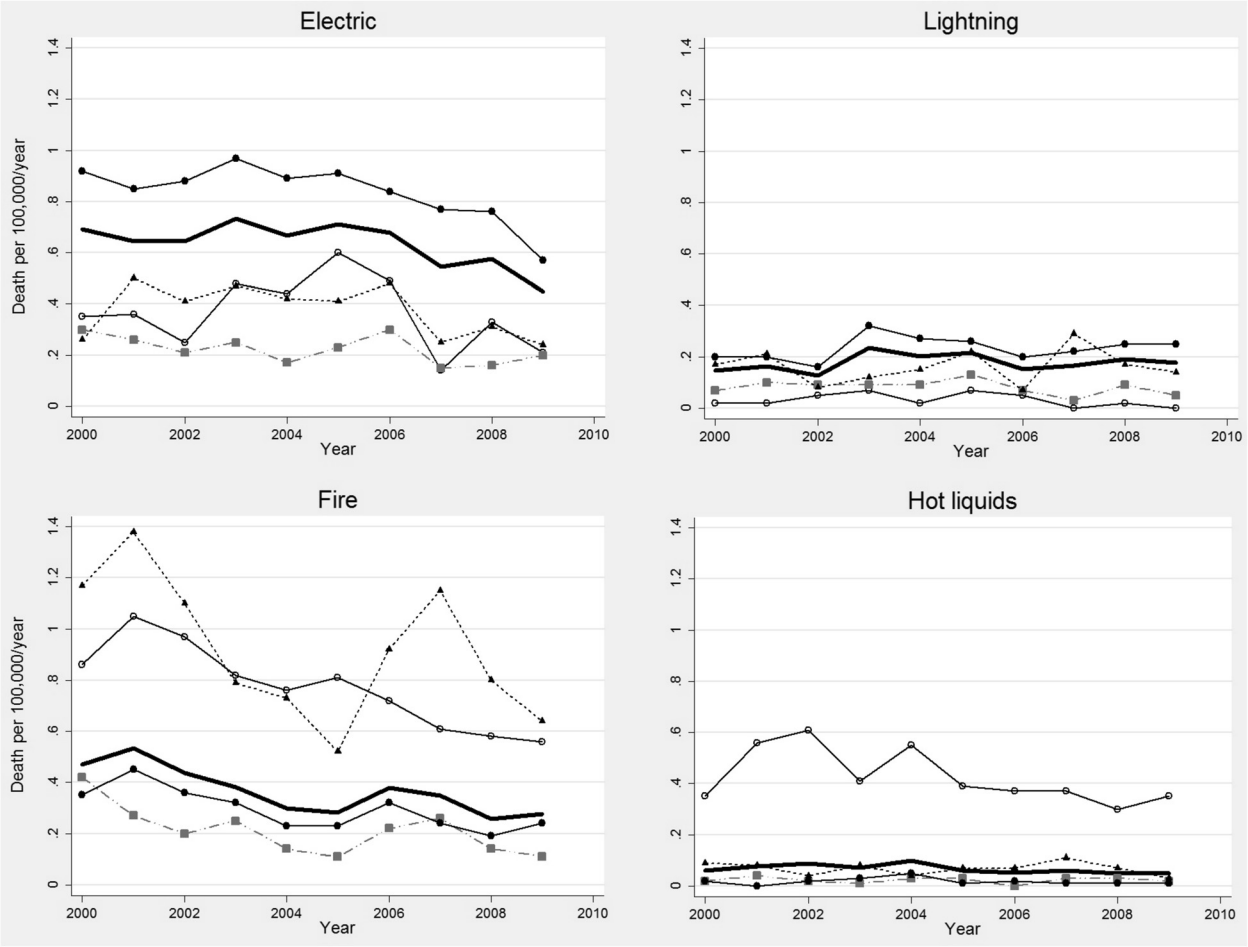
Mortality rates by cause of burns and age groups

Regarding the causal agent, it was found that electricity is the leading cause of deaths by burns in our country (49.5 %), followed by fire (28.5 %) and lightning injuries (13.9 %). The two electrical mechanisms (natural and human) were responsible for 4247 (78.0 %) of deaths during the study period (Table 3). However, there are differences according to the age. In the underage, the most common cause is injury due to fire (44.1 %), followed by electricity (29.9 %) and hot liquids (17.5 %). In adults, the most common cause is electricity (55.1 %), followed by fire (24.0 %) and lightning (15.8 %). Given this age difference, we present the frequency of burn agents by age group (Table 4) and mortality rates by cause of burns and age groups (Fig. 3).

**Table 3:** Mechanisms of injury in burns fatalities in Colombia

Burn agent	Deaths	%
Electric	2695	49.5
Lightning	757	13.9
Fire	1552	28.5
Hot liquids	283	5.2
Hot gases	23	0.4
Hot solid	20	0.4
Unspecified	36	0.7
Suicides	82	1.5

**Table 4:** Frequency and percentage of burn agents by age group (31 patients with indeterminate age)

Age group	0–5 years		6–14 years		15–64 years		>65 years	
	Deaths	%	Deaths	%	Deaths	%	Deaths	%
Electric	180	23.0	178	43.0	2225	58.6	98	23.1
Lightning	17	2.2	69	16.7	624	16.5	43	10.1
Fire	380	48.5	148	35.8	773	20.4	239	56.4
Hot liquids	193	24.6	17	4.1	54	1.4	18	4.3
Hot gases	3	0.4	1	0.2	15	0.4	4	0.9
Hot solid	3	0.4	1	0.2	13	0.3	3	0.7
Unspecified	7	0.9	0	0.0	20	0.5	9	2.1
Suicides	0	0.0	0	0.0	72	1.9	10	2.4
Total	783	100.0	414	100.0	3796	100.0	424	100.0

A percentage of 50.6 of those deceased (2759) did not receive medical attention during the process that produced their death. Regarding the site of death, 2093 (38.4 %) deaths happened in hospitals, clinics, or village health centers, 3238 (59.4 %) were outside health institutions, and there was no information on 117 (2.2 %). There are a significant number of deaths outside health institutions. As for the 3238 deaths outside hospitals, the main cause is electrical injury. Human and natural electricity (lightning) are the cause of 73.5 % of the deaths in households, 92.5 % of the deaths in the workplace, and 93 % on public roads. We present a description of the site of death according to the cause (Table 5).

**Table 5:** Frequency and percentage of burn agent according to the site of death (117 patients with indeterminate or unknown cause)

SITE	Hospital or clinics		Township or village health center		Home		Place working		Public road		Others	
Cause	Deaths	%	Deaths	%	Deaths	%	Deaths	%	Deaths	%	Deaths	%
Electric	551	26.8	30	73.2	723	53.6	452	69.2	553	79.0	313	58.3
Lightning	35	1.7	5	12.2	268	19.9	152	23.2	98	14.0	175	32.6
Fire	1069	52.1	6	14.6	339	25.1	45	6.9	36	5.1	42	7.8
Hot liquids	271	13.2	0	0.0	2	0.2	0	0.0	6	0.9	2	0.4
Hot gases	19	0.9	0	0.0	1	0.1	1	0.2	0	0.0	2	0.4
Hot solid	15	0.7	0	0.0	3	0.2	0	0.0	0	0.0	0	0.0
Unspecified	30	1.5	0	0.0	1	0.1	1	0.2	3	0.4	0	0.0
Suicides	62	3.0	0	0.0	11	0.8	2	0.3	4	0.6	3	0.5
Total	2052	100.0	41	100.0	1348	100.0	653	100.0	700	100.0	537	100.0

**Fig. 4:**
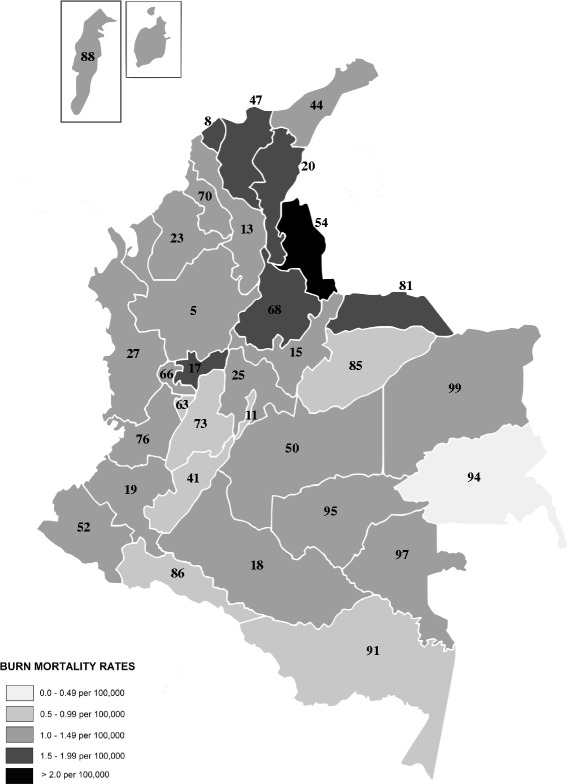
Topographic distribution of mortality rates of burns by departments in Colombia 2000 to 2009

In regard to the place of death, 3703 deaths (68.0 %) occurred in head municipalities, 441 (8.1 %) died in townships or small villages and jurisdictions, and 1264 (23.2 %) died in dispersed rural areas. There was no information on 40 certificates (0.7 %). The mortality rates were higher in rural area (1.55 per 100,000 per year) than the urban areas (1.17 per 100,000 per year). We obtained the number of deaths from counties and mortality rates were generated (Table 6). A political map was made by those counties (departments) indicating death rates in different colors (Fig. 4).

**Table 6:** Number of deaths and annual mortality rate from burns by county (department) of Colombia

Code	Department	Deaths	Average population	Annual death rate/100,000
05	Antioquia	696	5,643,511	1.23
08	Atlántico	402	2,151,045	1.87
11	Bogotá, D.C.	564	6,787,079	0.83
13	Bolívar	277	1,870,660	1.48
15	Boyacá	184	1,253,671	1.47
17	Caldas	162	967,591	1.67
18	Caquetá	59	418,052	1.41
19	Cauca	184	1,263,858	1.46
20	Cesar	152	897,334	1.69
23	Córdoba	184	1,457,229	1.26
25	Cundinamarca	235	2,260,293	1.04
27	Chocó	52	451,965	1.15
41	Huila	93	1,004,140	0.93
44	La Guajira	77	667,684	1.15
47	Magdalena	204	1,145,230	1.78
50	Meta	87	774,750	1.12
52	Nariño	174	1,531,949	1.14
54	Norte de Santander	285	1,238,465	2.30
63	Quindío	29	532,965	0.54
66	Risaralda	96	894,525	1.07
68	Santander	336	1,952,806	1.72
70	Sucre	87	768,273	1.13
73	Tolima	136	1,362,424	1.00
76	Valle del Cauca	570	4,139,615	1.38
81	Arauca	36	231,074	1.56
85	Casanare	26	292,197	0.89
86	Putumayo	24	308,638	0.78
88	Archipelago of San Andrés	9	70,237	1.28
91	Amazonas	5	67,277	0.74
94	Guainía	1	34,922	0.29
95	Guaviare	11	94,866	1.16
97	Vaupés	5	39,033	1.28
99	Vichada	6	55,188	1.09
	National total	5448	42,628,541	1.28

## Discussion

The WHO estimates that 195,227 people died in fires in 2008 across the world; the great majority happened in low-income and middle-income countries with a global mortality rate amounting to 2.9 per 100,000 per year [12]. The mortality rate in Colombia (1.28 per 100,000) is low compared to the global mortality rate and it is very similar to that of other countries such as Korea (1.3 per 100.000 in 2001) and the USA (1.09 per 100,000 in 2007) [13, 14] but far higher than that of Mexico (0.72 per 100,000 in 2007) [15]; though, our mortality rate has also been declining from 1.400 to 0.978 (year 2000 to 2009, respectively) as in other countries [15–18].

However, there is a difference with the data from international organizations. According to the WHO, Colombia has a burn mortality rate of 0.44 per 100,000 for 2008 similar to that of countries like Venezuela (0.43 per 100,000), Panama (0.29 per 100,000), Mexico (0.63 per 100,000), and Bolivia (0.38 per 100,000). According to this WHO data, 7752 deaths occurred in the Americas with a mortality rate of 0.85 deaths per 100,000 [12], which was well below that of Europe (2.3 per 100,000), Africa (4.9 per 100,000), and East and South Asia (4.8 per 100,000). Is our country and our region doing that well? This difference may have an explanation. Code U153 (GBD code) titled Unintentional injuries: Fires, used for the WHO reports, only includes the ICD-10 codes (X00–X09). It does not include codes for hot liquids (X10–X12), hot gases (X13, X14), hot solid (X15–X19), or electrical injuries (W85–W87) [19]. With electrical burns as our leading cause of death, the mortality rate in our study is 2.5-folds higher than that reported by the WHO. With the inclusion of other causes of burns, official mortality rates can be raised not only in our country but in countries with similar characteristics of development, showing that the problem is underestimated.

The results of this epidemiological study show male predominance of burns and compromise most productive ages. These results are consistent with several reports from other populations. It was observed that scalds are significantly more frequent in less than 5-year-old age group when compared with other causes of burns [18, 20–22]. Fire is a common cause of burns in children under 5 years old and men over 65 years old. Electricity affects mainly young workers so it is not surprising that there are populations with higher mortality rate than those under 5 years and over 65 years since the fatality rate in these populations is much higher.

In Colombia, in our experience, the higher frequency of electrical accidents is by accidental contact with power lines that pass very close to the houses, and they occurred usually, during local housing arrangement; in a few other cases, it is due to the illegal manipulation of electrical lines to obtain the wires and then sell the copper. Electricity can cause instant death even if the accident occurs at home with low voltage. Electricity generates cardiac arrhythmias, asystole, or prolonged respiratory arrest, which are fatal if resuscitation is not performed immediately. The vast majority die before being admitted to a medical institution [23–25].

It is interesting and surprising that lightning injuries are the third leading cause of death from burns. However, Colombia is reported as one of the countries with higher density of lightning in the world, probably because of its location relative to the equator in the inter-tropical confluence zone, the topographic variation, and its mountain ranges [26]. This can be explained by the high agricultural activity our country has; a quarter of our population live in remote rural areas, and there is lack of information on risk assessment and prevention.

There are other distinctive features of our burn mortality such as death at the scene without medical attention. Nearly half of those, who died from burns, died without getting pre-hospital medical care by trained personnel, relatives, or the community. This can be explained by two findings from our own study. First, almost all the people who suffer accidents in townships, villages, and dispersed rural areas die without medical care. Colombia is a country in northern South America; it has been estimated that by 2013, about a quarter of the population will be living outside urban areas. Colombia has a vast territory of 1,141,748 km^2^ which correspond to the mainland. It presents great topographic difficulty since there are three ridges which are the northern termination of the Andes. Secondly, of the accidents that occur in urban areas, a one third (32.0 %) is produced by electricity.

A limitation in our study is the difference between variables and categories during the years of the study, mainly after the implementation of electronic death certificates in 2008. At that time, new important information was added. It was impossible to determine variables, such as social security, education or marital status, or to identify whether the burn accident was related to the job. Only in the last 2 years of the study with the electronic format, the recording of deaths related to work, was included. Out of the 812 patients with the possibility of working, including children population, 144 had injuries related to the job (17.7 %). There is no such data for almost half of the cases (45.1 %). Another limitation is that there is very little availability of population-based studies to compare our findings with. Institution-based studies typically relate to the local population, the percentage of referrals from remote areas, the availability of hospital beds, the time from the accident to hospitalization, and pre-hospital fatality.

### Recommendations

Thus, the recommendations based on our results are as follow:

- To identify stakeholders and broader collaborative groups to work on prevention of burns;
- To create a national burn repository for epidemiologic and clinical data;
- To obtain data regarding the incidence of thermal injuries, the risk factors associated with burns in our community, and lethality;
- To generate prevention policies mainly in populations and areas of greatest risk (children, elderly, electrical safety);
- To implement multidisciplinary interventions for risk mitigation by community staff, primarily on the basic knowledge regarding first aid and resuscitation.

## Conclusions

Burn trauma is preventable in many cases. The first step towards the development of interventions for injury prevention is surveillance and identification of the risk factors. This is the first population-based burn death study in Colombia. But deaths are only the tip of the iceberg and the number of non-fatal injuries per year, morbidity, and lethality in our country is unknown.

Our study identifies risk at all ages for different reasons. Population groups with higher risk are those under 5 years and over 65 years old. The mortality rate tended to decrease during the length of the study and was higher in rural area than the urban areas. Electricity is the principal cause of burn deaths in Colombia, followed by fire and lightning and the deaths occurred mainly outside health institutions. Children under 5 years are affected principally due to fire and scalds.

A few specific recommendations can be given based on these epidemiologic features that will allow for the design of prevention strategies for this type of injury, focused mainly on electrical injuries in young people, fire burn injuries in children, and also first aid measures mainly regarding cardiopulmonary resuscitation.
